# The Child Delinquent

**Published:** 1916-04-08

**Authors:** Cecil M. Chapman


### THE CHILD DELINQUENT.

**An Address at a Meeting of the Child Study Society on March 30.**

By CECIL M. CHAPMAN, J.P.

Mr. Chapman said a delinquent was one who omitted a duty, and eventually committed faults and crimes. Among the people generally delinquent children were regarded as something worse than ordinary children, but that was not his own view. If asked for a definition he would say they were children with an unnatural propensity to break the laws of Society, which meant that, properly led, they would obey rather than break those laws, hence the duty of rulers was to find the causes which were operating. It had been authoritatively declared that delinquents, or those prone to evil, formed one-fifth of the children brought before magistrates. That proportion he considered high. Certainly quite two-fifths of the delinquents appearing at the Courts were the victims of neglect, either due to their parents or to the environment in which they were brought up. That being so, a good case existed for the present-day refusal to treat these children as if they were criminal. In tracing the evolution towards this larger and better view, he reminded the meeting that in 1833 a boy aged nine was condemned to death for stealing two-pennyworth of paint, a sentence which was subsequently commuted to transportation for life. Severe punishment had proved to be a great encouragement to crime, because people neglected to charge and juries to convict when they knew what would follow, hence many offences went unnoticed by the Courts. There was now scarcely any authority who believed that imprisonment, with or without hard labour, did good in anything like the majority of cases which it was supposed to suit. By the use of the First Offenders Act and the Probation Act magistrates were able to distinguish between the habitual offender and the first offender; an effort was made to discover whether the person was a criminal, or whether the act was an isolated one due to a sudden impulse or temptation. That this was wise became evident from the fact that while the criminal statistics of some European countries were getting worse, the British figures were improving.

As to causes of criminality in children, malnutrition tended to moral weakness in the presence of temptation, and the contributory parental incompetency and neglect were very common. He had often been told that this was largely due to the stress of life. Bad environment, such as in the slum, with no opportunities for real recreation, was not only all against the child, but he considered it a miracle that the products of these circumstances were as good as they were. Some people's souls, even the tenderly brought up, were so small that they needed not only fostering, but even intensive culture to keep them straight. A very important necessity for the proper evolution of the child was a real respect for both parents. He believed that in great cities respect was as rare as was the grace of God in a gin-shop, as Americans put it. And the great part played by the child's imitative faculty should never be lost sight of. He considered that with an improvement in our national system of education children might have a greater communal sense and a higher *esprit de corps*, and so realise the duties each owed to the rest.

He had no doubt whatever that recently, and especially since the war commenced, criminality, particularly among children, had much increased: between November and January the increase was 40 per cent., and almost entirely in the matter of theft. Two contributory factors were the darkened streets, and the absence of the male parent on service. Among adults there was noticeable a great moral laxness when the property of the Government, railway companies, or large combines was concerned. A bad class of case was that in which parents sent their children into shops or stores to steal. The children designated "beyond control" were often found to be those whose parents had married again, and among these were many whose offence was sleeping out—under vans, or in railway arches—rather than go home to their beds. These children seemed to him to have a good deal of real courage, and it was probably the sort of material out of which Arctic explorers and aeroplanists were made. The root cause of the cases of begging, in some instances, was the attraction of the cinema houses, but in many cases he was satisfied that the children were really hungry, their state was beyond that called malnutrition. Those children needed to be saved from their parents, and sometimes that was effectually accomplished. Wilful damage and sexual offences were both uncommon among children. Everyone should realise the duty of making human nuisances into human citizens of value. Industrial schools and reformatories were doing much, but an urgently needed reform was the raising of the age from sixteen to at least seventeen years, and, in the case of girls, the "age of consent" should be raised to eighteen. There was need to penetrate the "Englishman's castle" in order to know the horrors which were going on within; and he strongly advocated the appointment of more women inspectors and women industrial officers.

